# Complications and Sequels of Prostatectomy

**Published:** 1904-04-30

**Authors:** 


### COMPLICATIONS AND SEQUELS OF PROSTATECTOMY.

In view of the growing inclination that appears to exist towards recommending patients suffering from enlarged prostate to undergo radical treatment without waiting for the more serious symptoms to appear, it is well to consider what are the drawbacks to the operation. It is clear from the situation of the organ that its removal is not and never can be a trivial affair. Although the immediate mortality in careful hands is small, yet it is far from negligable, and, moreover, in a certain percentage of those who recover complications and sequelæ are almost certain to arise and detract from the success of the operation. In discussing this aspect of the matter Dr. J. E. Moore[1] points out that the most frequent cause of death following radical treatment is uræmia, consequently the most important preliminary examinations are those of the kidneys. Unfortunately, the enlarged prostate and inflammation of the bladder render it difficult, or impossible, to ascertain the condition of the kidneys; but the surgeon is justified in operating so long as there is no positive evidence of renal complications. The next greatest danger is sepsis, and in the presence of an infected bladder it is an ever-present one. The patients in whom the operation is required usually have not so much resistance against wound infection as younger men. All that can be done to counteract this is to thoroughly cleanse the wound, and to provide free drainage. The complication of cystitis is often at the present time the most positive indication for operation, and the only satisfactory treatment for it is drainage. Stone is a frequent complication of enlarged prostate and should always be sought for during prostatectomy. Cases are on record in which a calculus has been overlooked, and as a consequence the operation has been a failure. Such a mistake is by no means unlikely in perinaeal prostatectomy unless a deliberate search is made for stone. The incision, when too extensive, may cause various complications. In the suprapubic operation it may injure the peritoneum, and in the perinaeal one important muscles, blood-vessels, and nerves may be damaged. Ordinarily the peritoneum is easy to avoid, but with a contracted bladder the danger is great. Hæmorrhage is not a common complication; when it does occur it is best controlled by ligature or forcipressure, as packing causes severe pain and may produce sloughing.

Too little effort is made, Dr. Moore believes, to protect the urethra, especially in operating by the perinaeal route. It simplifies the operation wonderfully to sacrifice the whole prostatic urethra, as is frequently done; and it is marvellous how quickly nature compensates for its loss; but it is not reasonable to suppose that traumatic stricture will not occur later, and a man with a traumatic stricture at the neck of his bladder may be in a worse condition than one with an enlarged prostate. The most difficult problem in the perinaeal operation is to avoid injuring the rectum. There are few, if any, operators who have never met with this accident. It is not a disastrous event when it does happen, though it is always a serious complication. There is another drawback to prostatectomy as usually performed, namely, the sacrifice of the seminal ducts. An occasional sequel of removal of the prostate is dribbling of urine owing to injury of muscles or nerves at the neck of the bladder, but the condition can be avoided by care and gentle operating. A perinaeal fistula calling for a plastic operation is another occasional sequel, and may be caused by too protracted draining. It is rarely necessary to keep a tube in the bladder for more than a week. Epididymitis is a quite frequent consequence of the operation, and may become so troublesome as to require removal of the epididymis. Dr. Moore's opinion is that the operation of prostatectomy is altogether too grave a one to be resorted to as a routine treatment for every enlarged prostate.

[1] Annals of Surgery, April 1904.

